# Primary health care assessment in Brazil: conception and methodology of the 2024 National Basic Health Unit Census

**DOI:** 10.1590/0102-311XEN164625

**Published:** 2026-04-10

**Authors:** Aylene Bousquat, Ligia Giovanella, Rosana Aquino, Ana Luiza Queiroz Vilasbôas, Elaine Thumé, Elaine Tomasi, Maria Helena Magalhães de Mendonça, Alaneir de Fátima da Silva, Patty Fidelis de Almeida, Paulo Henrique dos Santos Mota, Carlos Pilz, Elisandréa Sguario Kemper, Dirceu Klitzke, Gabriela da Silva Formoso, Bruna Venturin, Luiz Augusto Facchini

**Affiliations:** 1 Faculdade de Saúde Pública, Universidade de São Paulo, São Paulo, Brasil.; 2 Centro de Estudos Estratégicos da Fiocruz Antonio Ivo de Carvalho, Fundação Oswaldo Cruz, Rio de Janeiro, Brasil.; 3 Instituto de Saúde Coletiva, Universidade Federal da Bahia, Salvador, Brasil.; 4 Faculdade de Enfermagem, Universidade Federal de Pelotas, Pelotas, Brasil.; 5 Faculdade de Medicina, Universidade Federal de Pelotas, Pelotas, Brasil.; 6 Escola Nacional de Saúde Pública Sergio Arouca, Fundação Oswaldo Cruz, Rio de Janeiro, Brasil.; 7 Faculdade de Medicina, Universidade Federal de Minas Gerais, Belo Horizonte, Brasil.; 8 Universidade Federal Fluminense, Niterói, Brasil.; 9 Secretaria de Atenção Primária à Saúde, Ministério da Saúde, Brasília, Brasil.; 10 Centro de Integração de Dados e Conhecimentos para Saúde, Salvador, Brasil.

**Keywords:** Primary Health Care, Health Evaluation, Health Services, Health Services Research, Atención Primaria de Salud, Evaluación en Salud, Servicios de Salud, Investigación sobre Servicios de Salud

## Abstract

Primary health care (PHC) is the basis of the Brazilian Unified National Health System (SUS). After a discontinuity in PHC evaluation policies, the Ministry of Health, with the support of the PHC Research Network of the Brazilian Association of Public Health (Abrasco), carried out the Brazilian National Basic Health Unit Census in 2024. The objective of this article is to present its creation and operationalization. The census methodology was based on a participatory approach, involving several actors, including departments of the Brazilian Ministry of Health, the Brazilian National Council of Health, the Brazilian National Council of Municipal Health Departments, academic institutions, and the Brazilian National Health Council. The data collection instrument was built collaboratively and applied online, using the e-Gestor PHC platform to ensure the institutionalization and security of the data. Nationwide, the operationalization received support in all states, mobilizing managers and professionals. All municipalities adhered to the census, with responses from all 44,938 basic health units (BHU) in Brazil. This success, despite the methodological challenges inherent to online surveys, demonstrates the engagement of the actors involved in strengthening the SUS. The process of preparing the 2024 BHU Census encompasses elements that make it an essential tool to the formulation of more effective, equitable public policies aligned with the principles of PHC and SUS, contributing significantly to the institutionalization of PHC evaluation in Brazil.

## Abstract (es)

La atención primaria de salud (APS) es la base del Sistema Único de Salud (SUS). Tras un período de discontinuidad en las políticas de evaluación de la APS, el Ministerio de Salud brasileño, con el apoyo de la Red de Investigación en APS de la Asociación Brasileña de Salud Colectiva (Abrasco), llevó a cabo el Censo Nacional de Unidades Básicas de Salud en el 2024. El objetivo de este artículo es presentar su concepción y operativización. La metodología del censo se basó en un enfoque participativo con la colaboración de diversos actores, entre ellos las secretarías del Ministerio de Saud brasileño, el Consejo Nacional de Secretarios de Salud, el Consejo Nacional de Secretarías Municipales de Salud, instituciones académicas y el Consejo Nacional de Salud. El instrumento de recopilación de datos se construyó de forma colaborativa y se aplicó en línea, utilizando la plataforma e-Gestor APS para asegurar la institucionalización y la seguridad de los datos. La operativización contó con una amplia red de apoyo en todos los estados, con el objetivo de movilizar a gestores y profesionales. El censo logró una adhesión del 100% de los municipios, con respuestas de todas las 44.938 unidades básicas de salud (UBS) del país. Este éxito, a pesar de los desafíos metodológicos inherentes a las encuestas en línea, refleja la dedicación de los actores comprometidos con el fortalecimiento del SUS. El proceso de elaboración del Censo de las UBS 2024 incluye elementos que lo cualifican para contribuir a la formulación de políticas públicas más efectivas, equitativas y alineadas a los principios de la APS y del SUS, lo que contribuye significativamente a la institucionalización de la evaluación de la APS en Brasil.

## Introduction

Primary health care (PHC) is internationally recognized as the cornerstone of health care systems, being essential to ensure universal access, coordinated care, and comprehensive service ^1^ ^,^ ^2^. Over three decades, the Family Health Strategy (FHS) has consolidated as the main model for organizing PHC in Brazil. This model helped reduce health care inequalities and improve morbidity and mortality indicators. Brazilian PHC assessments in recent decades have shown its contributions toward the achievement of the Brazilian Unified National Health System (SUS, acronym in Portuguese) constitutional principles: universality, integrality, and equity ^3^ ^,^ ^4^ ^,^ ^5^ ^,^ ^6^ ^,^ ^7^.

In the international context, PHC assessments have informed the design of public policies and promoted quality health care. Notable initiatives include the Quality and Outcomes Framework (QOF), implemented in the United Kingdom since 2004, establishing pay-for-performance mechanisms and standardized quality indicators ^8^. In the Americas, the Pan-American Health Organization (PAHO), in partnership with the World Bank, leads the Primary Health Care Measurement and Improvement Initiative (PHCMI), which aims to standardize and support PHC assessment systems in member countries ^9^.

In Brazil, a continent-sized country with the largest public PHC service network in the world, PHC assessment is not a trivial task, requiring participatory and innovative models. Furthermore, the expanded conception of Brazilian PHC - not restricted to the first level of outpatient care, but structured with a strong community and territorial dimension, and with functions such as care coordination, by continuously monitoring each person’s clinical trajectory, and such as a health care network organizer, by organizing flows and regulating access between the different SUS services - poses new and necessary evaluative challenges ^1^ ^,^ ^10^ ^,^ ^11^.

Understanding the need to support the assessment of Brazilian PHC, the Ministry of Health, via the Secretariat of Primary Health Care (SAPS, acronym in Portuguese), with support from the PHC Research Network of the Brazilian Association of Collective Health (Rede APS/Abrasco, acronym in Portuguese), conducted the Brazilian National Basic Health Unit Census (UBS Census) in 2024. The BHU Census marks the resumption of a PHC assessment policy for all basic health units (BHU), after a 12-year period. Notably, the 2012 census survey under the Improvement Access and Quality National Program of Primary Health Care (PMAQ-AB, acronym in Portuguese) only collected structural information for the BHU set. Therefore, the 2024 BHU Census’ current format - with PHC service infrastructure and provision data - represents an unprecedented process as to the scope and characterization of Brazilian BHUs. In addition to updating the database on the PHC service network and its operating conditions, the census was aimed at providing inputs to guide public health care policies, supporting not only specific decision-making but also contributing toward a national assessment and monitoring policy. Its design was guided by the SUS principles, in favor of the universalization and qualification of the FHS, providing a detailed situational analysis of Brazil’s PHC. This article aims to present the conception and operationalization of the 2024 BHU Census. The census development process was based on two complementary and inseparable goals: (1) ensuring a process that was consistent with the Brazilian federative structure and (2) defining the target image of the Brazilian PHC, understood as integral, effective, community-based, territorial, and fully integrated into the health care service network. 

## The 2024 BHU Census and the Brazilian PHC assessment 

In the last three decades, Brazil’s government has implemented several PHC performance monitoring and assessment initiatives, with a special emphasis on the FHS. In 1998, the Primary Health Care Information System (SIAB, acronym in Portuguese) was established for routine monitoring of team activities. In the same year, the Primary Health Care Indicators Pact was created, nationally consolidating specific PHC monitoring instruments. In 2002, the launch of the Family Health Expansion and Consolidation Project (PROESF, acronym in Portuguese) provided significant advances as to assessment and monitoring, including by fostering assessment research, and, in 2005, the Baseline Studies established the initial performance indicators ^12^. 

In 2006, the Brazilian National Primary Health Care Policy (PNAB) unprecedentedly incorporated a formal inter-federative goal-setting process, through the Primary Health Care Indicators Pact, as a strategy for monitoring PHC performance and inducing improved health care quality. This pact provided for the definition and annual monitoring of health indicator goals previously agreed-upon by the federative units, in compliance with the Pact for Health. The achievement of these goals was associated with the annual correction of the Fixed Primary Health Care Floor (PAB Fixo, acronym in Portuguese) values, thus establishing a direct relation between performance and federal funding. This initiative represented a milestone in the institutionalization of PHC performance assessment in Brazil, promoting higher co-responsibility between government spheres and promoting the consolidation of PHC as a strategic public policy in the SUS. In this same period, the Pact for Health introduced new team management and assessment parameters, followed, in 2011, by the implementation of the quality improvement assessment, which introduced the measurement of qualitative and organizational aspects of FHS teams. 

The PMAQ-AB was another important process in PHC assessment in Brazil ^13^. In 2012, within the scope of the PMAQ-AB, there was the first Brazilian National BHU Structure Census, providing a comprehensive survey of the structural conditions of the units ^14^. Subsequent PMAQ-AB assessment cycles were carried out in 2012-2014, 2015-2016, and 2017-2018, analyzing the health care teams, especially family health teams (FHT), oral health yeams (OHT), and Family Health Support Nucleus (NASF, acronym in Portuguese).

Periodic infrastructure, care practice, work organization, and user satisfaction data collections during this period produced a comprehensive database that supported strategic decision-making in national public policies ^15^ ^,^ ^16^.

The period following the last PMAQ-AB cycle (2017-2018) was marked by: increasing institutional weakening in health care policies; program discontinuities; dismantling of technical teams; and loss of federal mechanisms for induction and monitoring in general and particularly for PHC ^10^ ^,^ ^11^. In 2019, previous assessment processes were replaced with the proposal for quarterly monitoring of seven health team performance indicators, within the pay-for-performance component of the Previne Brasil program. The Previne Brasil program was a precarious and restricted solution for continuity in the PHC assessment policy then in force, contributing to the dismantling of PHC assessment and institutionalization ^17^ ^,^ ^18^.

The BHU Census, conducted in 2024, marks the resumption of a national assessment and monitoring policy, based on the PHC and SUS principles, contributing toward the institutionalization of assessment in the SUS, as will be demonstrated below.

**Figure 1:**
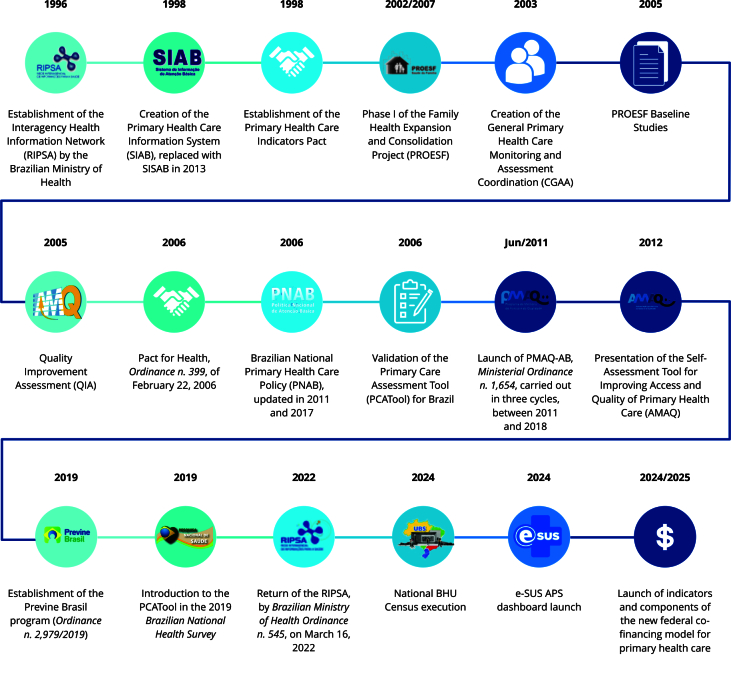
Key public health policy monitoring and assessment milestones in Brazil.

Figure 1 shows the timeline for the main health care policy monitoring and assessment milestones between 1996 and 2025.

PMAQ-AB: Improvement Access and Quality National Program of Primary Health Care. Source: prepared by the authors.

## Methodology

We adopted the following methodological steps: census governance; universe definition; questionnaire development and validation; data collection; census operational strategies; census participants and database completeness analysis; limitations; and ethical aspects.

### Census governance

The methodological development of the BHU Census was based on the participatory approach of the fourth-generation assessment proposed by Guba & Lincoln ^19^. This model emphasizes the active participation of different groups engaged in the construction of the assessment instrument, prioritizing the negotiation of meanings and the collective construction of the study’s dimensions and indicators. This approach was adopted to ensure the relevance, legitimacy, and representativeness of the assessed aspects, considering the regional specificities and concrete needs of Brazilian PHC local teams and managers. 

Accordingly, in addition to SAPS and the Rede APS/Abrasco, the process actively included: other departments of the Brazilian Ministry of Health; the Brazilian National Council of State Departments of Health (CONASS, acronym in Portuguese), representing state health departments; the Brazilian National Council of Municipal Departments of Health (CONASEMS, acronym in Portuguese), representing municipal health departments; academic and research institutions; professional representative entities; international organizations, such as the PAHO, and; the Brazilian National Health Council (CNS, acronym in Portuguese), representing social control and community participation. 

### Universe definition

Despite the existence of the Brazilian National Registry of Health Establishments (CNES, acronym in Portuguese), defining the study universe was a complex task. As the goal was to include all health units that offered PHC, several units that offered outpatient care for specific groups were excluded, such as pediatric care and gynecology. Notably, these were often listed as PHC in the CNES. Furthermore, several units that at first were BHU, changed their activities but remained registered as PHC. Thus, the following sequential steps were taken to obtain the universe: (i) inclusion of all units listed as PHC in the CNES in April 2024; (ii) validation by municipal managers; (iii) checking of census responses.

### Questionnaire development and validation

The preliminary instrument, developed by the Rede APS/Abrasco research team, was guided by the concept of community-oriented, territorialized, quality, and effective PHC, integrated into the regionalized health care network of the SUS ^20^ between May and September 2023. This comprehensive approach is based on multidisciplinary teams with interprofessional and collaborative work; territorial and community focus; health surveillance and accountability for the health of the population in a given territory; social participation and intersectorality; expanded scope of individual and collective actions; care coordination and integration into the health care network; intercultural approach; promotion of equity; and valuing of the workforce. All these aspects were included in the questionnaire. The team that developed the first version of the instrument was composed of 11 senior researchers with extensive experience in PHC assessment. Whenever relevant, PMAQ-AB questions were retrieved, which, with due methodological care, would enable comparisons. To ensure that the final instrument would be capable of both reflecting the needs and specificities of PHC in the country and planning its operationalization, several technical workshops and meetings were held. These included the participation of numerous stakeholders, starting with the first version of the questionnaire. Between September 2023 and February 2024, four national in-person workshops were held focusing on the initial instrument, making adjustments until the instrument was finalized for pre-testing. Weekly technical meetings for instrument improvement were held between September 2023 and March 2024 and served as spaces for reaching consensus.

During the last national in-person workshop, participants identified the main challenges for census implementation and proposed solutions to overcome them. The challenges notably included the need to ensure instrument reliability and objectivity, mobilize and engage the actors involved, overcome technological bottlenecks, consider political and temporal aspects, and ensure research confidentiality and ethics. 

The proposals to overcome these challenges involved reviewing and improving the data collection instrument and developing its completion instruction manual, developing communication and mobilization strategies, training respondents, implementing technical and logistical support, as well as planning an adequate schedule.

Finally, the BHU Census instrument, proposed after the rounds of technical workshops and meetings, underwent a validation process that included a new review by specialists, pilot testing, and tripartite agreement. The pilot tests were initially implemented with a paper-printed questionnaire in six BHU chosen for specifically analyzing the respondents’ understanding of the questions, as well as to observe its feasibility in different types of BHU, such as, for example, with and without FHS. In May 2024, a new pilot test was conducted with an online instrument in BHU in three states of Brazil, in order to trace difficulties in using the platform. After the two tests, we made minor adjustments that did not substantially modify the instrument.

The final questionnaire aggregated 15 dimensions and 141 questions, with 140 close-ended, Likert scale, and a smaller number of ordinal responses, in addition to an open-ended question included at the end of the instrument as optional for respondent comments as desired. Each dimension was divided into specific components, covering detailed information on physical infrastructure, organization and delivery of service, care practices, care coordination, technological incorporation, team management and work processes, community and intersectoral actions, health surveillance, and measures aimed at promoting equity, professional appreciation, among others (Box 1). To ensure consistency with the distinct regional characteristics, we included questions concerning ribeirinhos teams, as well as other traditional populations and peoples. More information and details on dimensions and variables are available in the questionnaire ^21^ and in the instructional manual ^22^ from the Brazilian Ministry of Health.

**Box 1:** Dimensions and components evaluated in the Brazilian National Basic Health Unit Census, 2024.

DIMENSION	COMPONENTS
1. BHU Identification	Location, operation, and administrative management data
2. Composition of PHC teams, management, and work process	Types and number of teams; professionals working at the BHU; team work process; types of employment relationships and contracts
3. BHU infrastructure conditions	Physical structure and ambiance; accessibility; availability of equipment and supplies; construction, expansion, and renovations
4. Digital health care: incorporation of digital technologies and telemedicine	Information technology equipment and internet access; telemedicine; use of electronic health records
5. Availability and access to diagnostic methods and medications	Performance of diagnostic exams and tests; access to medications
6. Availability of actions and services at the BHU: scope of team practices	Sexual and reproductive health; prenatal and postpartum care; detection, prevention, and screening of cervical and breast cancer; care for children up to two years of age; care for individuals with systemic arterial hypertension; care for individuals with diabetes mellitus; care for individuals with overweight/obesity; care for individuals with tuberculosis; care for individuals with leprosy; mental health care; care for individuals experiencing violence; care for the health of the older adults; comprehensive men’s health care; care for bedridden individuals; promotion of universal vaccination coverage; care for post-COVID-19 conditions; integrative and complementary practices (PICS); care for urgent and emergency situations
7. Health promotion and intersectoral actions	Health promotion actions and programs; Health Academy Program; School Health Program (PSE); *Bolsa Família* (Brazilian Income Transfer Program) and intersectoral actions
8. Health surveillance	Epidemiological surveillance activities; food and nutritional surveillance; sanitary and environmental surveillance; control of *Aedes aegypti*
9. Oral health teams: practice and scope	Organization of the team's service schedule and availability; relation of the oral health team with the health care network; oral cancer care
10. CHW practice	Specific activities of CHW
11. Care coordination and integration into the health care network	Schedule organization, accommodation and spontaneous demand; integration of PHC with other points in the network; health care regulation and sanitary transport
12. Shared care and practice of multidisciplinary teams in PHC	Composition and scope of multidisciplinary teams
13. Community and territorial actions	Territorialization and reference population; social participation and user-related communication strategies
14. Promotion of equity	Care for traditional populations and specific groups; care for people with disabilities; actions to promote gender, sexual, ethnic, and racial equity
15. Continuing education, qualification, and valorization of workers	Actions related to worker health and labor management

BHU: basic health units; CHW: community health workers; PHC: primary health care.

Source: prepared by the authors.

### Data collection

Based on the previous surveys conducted by the Rede APS/Abrasco during the COVID-19 pandemic ^23^ ^,^ ^24^, the online format was chosen for data collection. Note that online surveys are faster and demand lower financial investment than in-person versions, qualities that are extremely desirable in public administration. 

To facilitate and ensure the institutionalization of the process, we chose to use of the Brazilian Ministry of Health’s official information systems, which, in addition to ensuring data traffic security, is routinely used by municipal departments and BHU. Thus, we used the *e-Gestor Atenção Primária à Saúde* (e-Gestor APS), a computerized platform developed by SAPS for managing registrations of establishments, teams, and professionals, as well as for monitoring federal programs and fundings within the scope of PHC. The system was accessed through authentication via gov.br account, the official digital identification mechanism of the Federal Government, as provided for in the access protocols of the e-Gestor APS system itself ^25^. This configuration ensured nominal identification of users and traceability of actions on the platform, ensuring records integrity. Within the scope of the BHU Census, e-Gestor APS was used as the exclusive operational environment for census management. 

Initially, the municipal manager should indicate their decision to participate in the BHU Census, through a specific feature made available on the *Gerencia APS* platform, requiring prior authorization and authentication. Subsequently, there was registration and designation of the municipal responsible for the BHU Census. After joining, the municipal manager accessed a list that included PHC facilities based on data available in the CNES in April 2024. In case any of these facilities were no longer operating, or were no longer truly a PHC service, they should be deactivated. For each active service, the census manager would indicate a respondent to fill out the census questionnaire.

After the person responsible for filling out the census questionnaire was indicated, the data collection instrument was made available electronically through the LimeSurvey platform (https://www.limesurvey.org/pt-br), hosted on the Brazilian Ministry of Health’s infrastructure and integrated into e-Gestor APS. 

The process of joining in and subsequently completing the questionnaires began on June 3, 2024 and concluded on September 30, 2024, with a specific extension for municipalities in Rio Grande do Sul due to exceptional climatic events faced by the state's population. Thus, census results refer to BHU profiles between June and September 2024.

### Census operational strategies

To ensure adherence and responses, we structured a broad support network, composed of specific teams in each state, which was responsible for institutional coordination, strategic planning of local actions, and supervision of activities, as well as support for data collection. The teams , organized by the Rede APS/Abrasco, consisted of: one senior researcher; one to four supervisors/facilitators, in addition to one to 12 animators. The constitution of field work teams in each state aimed to favor communication and develop strategies for adherence and participation of municipal managers, local managers, and professionals of basic health units in filling out the census instrument, with coordination, cooperation, and mobilization activities reflecting the adopted governance concept.

The state census coordinators established institutional contacts and promoted periodic meetings, as well as joint work with representatives from the State Health Departments (SES, acronym in Portuguese), the Municipal Health Secretariat Councils (COSEMS, acronym in Portuguese), and the State Commissioner of the Ministry of Health, ensuring the necessary institutional support for conducting the census. Furthermore, they regularly participated in meetings of the Bipartite Inter-manager Commissions (CIB, acronym in Portuguese) and Regional Inter-manager Commissions (CIR, acronym in Portuguese), in which they formalized essential agreements for broad adherence to the census.

The animators were tasked with making direct and systematic contact with municipal managers and health teams of the BHU, ensuring adherence to the census and clarifying operational doubts related to filling out the questionnaire. Contacts were predominantly via telephone, messaging apps, institutional e-mails, and video conferences. In cases of difficulties with joining in or answering the census, animators organized online and/or in-person meetings to provide direct support and resolve operational issues. Due to the large volume of data, at various times, there was platform instability, which demanded greater support for BHU teams. For the management and monitoring of daily activities, each state team built its own instruments, with the use of shared spreadsheets being noted. These documents recorded daily contact attempts, the detailed status of municipal adherence, the identification of local professionals responsible for responses, and any difficulties encountered. The teams received daily reports produced by the Brazilian Ministry of Health team with the response status of each facility participating in the census in their territory. This organization provided continuous monitoring and rapid, targeted interventions by state supervisor whenever necessary.

The state facilitators, in joint action with SAPS technicians, promoted several initiatives to integrate the actors. In several states, there were partnerships with COSEMS with coordinated joint action, including their supporters, for dissemination of the census and encouragement of adherence. In partnership with state PHC supervisor committee of the SES, there was mobilization of state PHC supporters; regional SES supporters in the CIRs with participation of state facilitators and animators in CIR meetings. This set of strategies and actors helped make the census feasible.

As a complementary mobilization and clarification strategy, we held informative digital events, such as live streams and webinars, specifically targeting municipal managers and BHU teams. These events had the specific objective of raising the participants’ awareness about the relevance of the study and offering detailed guidance on the methodological and operational procedures for completing the questionnaire.

In order to expand the possibilities for clarifying doubts in a more interactive manner, we released support materials such as manuals and instructional videos detailing the step-by-step process for expressing interest, registering respondents, and answering the census. The instructional videos and national live streams held by Rede APS/Abrasco, the Brazilian Ministry of Health, and CONASEMS addressed different stages of the census and reached 29,500 views, not to mention that there were some state live streams, such as the one in São Paulo, which had 2,500 accesses. Online technical meetings were held in all states with the participation of SAPS, CONASS, CONASEMS, COSEMS, Rede APS/Abrasco, facilitators, and animators.

Finally, to ensure streamlined operational decisions and effective communication between the various state teams and the national census coordination, institutional groups were organized through a messaging application. These groups included state supervisors, representatives from the SES, COSEMS, and the national team of the Brazilian Ministry of Health. This tool enabled efficient resolution of operational problems, rapid dissemination of best practices identified in different states, and reinforced methodological alignment throughout the national territory.

Still from the perspective of ensuring support and clarification on any doubts or difficulties related to the census platform, we structured a service environment that included the following channels: *Disque Censo*, via WhatsApp and telephone service, available during business hours, a specific e-mail, and a web service of the e-Gestor APS system. After the first two weeks of the census data collection, we created a Frequently Asked Questions (FAQ) section containing answers to the main questions received through the service channels ^26^. These resources offered streamlined and precise guidance to health managers and professionals, ensuring greater efficiency in completion, guaranteeing that doubts or problems encountered were answered as quickly as possible.

### Census participants and database completeness analysis

**Figure 2:**
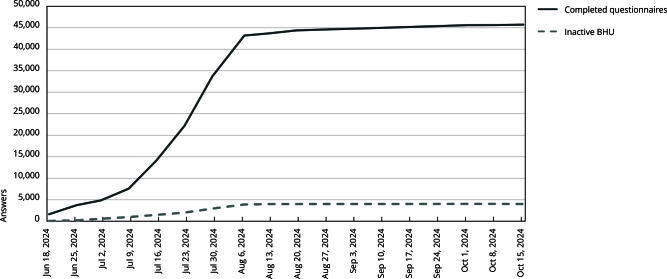
Evolution of census answers during the execution period.

The BHU Census was census-based, cross-sectional, and descriptive, and had 100% adherence from municipal managers. Initially, it included 49,738 health care facilities registered in the CNES in the SUS primary health care services category in April 2024. These services encompassed over 90,000 primary health care teams (PHCT), FHT, OHT, multidisciplinary teams, and other team modes, such as street clinic teams (SCT), prison primary health care teams (PPHCT), and family health teams for *ribeirinho* communities (RFHT). Of this total, 4,026 facilities were deactivated by municipal managers, as they were not in operation at the time, or because they were another type of facility not related to PHC. Figure 2 shows the evolution of the answers during the period the questionnaire was available for completion. 

BHU: basic health units. Source: prepared by the authors.

**Figure 3:**
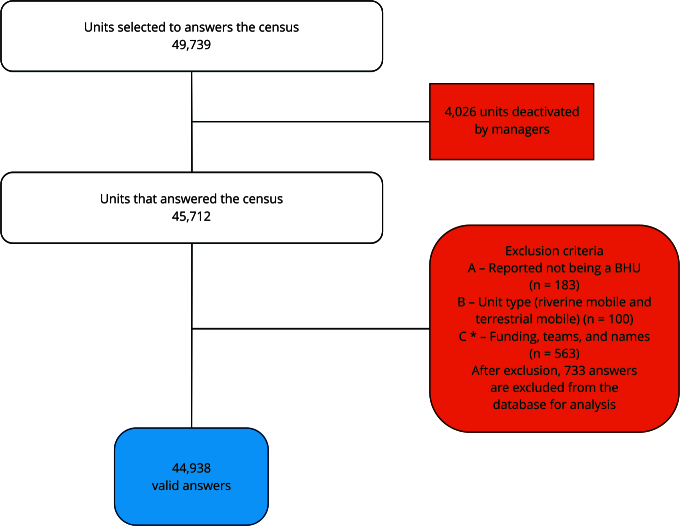
Flowchart of the number of answers obtained by the census.

In total, 45,712 health care facilities answered the census. The database was analyzed as to completeness and consistency, and it was observed that, even after the inactivation process, some facilities that were not PHC units had answered the questionnaire. Thus, we excluded from the analysis establishments that: (a) informed, in an open-ended question, that they were not PHC facilities; (b) land or river mobile units; (c) lacked funding from the Brazilian Ministry of Health for FHT, PHCT, RFHT, and community health workers (CHW) in the 12 months prior to the census, and declared lacking FHT, PHCT, and RFHT, in addition to having names clearly indicative of establishments of another level of care (Covid Center, Birthing Centers, Specialty Centers, Dental Specialty Centers, Rehabilitation Centers), with the exclusion of 773 facilities (Figure 3).

BHU: basic health units. * Units lacked Brazilian Ministry of Health funding for family health teams (FHT), primary health care teams (PHCT), family health teams for *ribeirinho* communities (RFHT), and community health workers (CHW), and declared lacking FHS, PHCT, and RFHT, in addition to having names clearly indicative of a facility of another level of care. Source: prepared by the authors.

Thus, the universe comprised 44,938 Brazilian BHU, with the following distribution across the country’s regions: North = 4,096 (9.11%), Northeast = 17,737 (39.47%), Southeast = 13,375 (29.76%), South = 6,607 (14.7%), and Central-West = 3,213 (6.95%) facilities.

### Limitations

The literature indicates high rates for dropout or loss of responses in online studies, causing selection bias and lack of representativeness of the sample ^27^ ^,^ ^28^. In the case of the census, there was evidently no sampling design, but losses could have occurred for the most diverse reasons, from internet connection difficulties to the absence of qualified professionals, leading to selection biases. This scenario did not occur, and these challenges were overcome, with all BHU in the country completing the instrument. 

Despite this success, the methodological limitations and challenges faced cannot be overlooked. After the initial database completeness analysis steps, a few questions with inconsistent answers were identified. We decided to exclude such variables, namely: the opening and closing hours of the BHU on Saturdays and Sundays, and the question regarding the responding unit being a Support Point. According to the Brazilian Ministry of Health ^29^, the Support Point, despite having its own CNES, is linked to a BHU with which it periodically shares the health care team, in such a way that its activities, especially those of higher-level professionals, are not daily, but this was not clear to the respondents. 

It is noteworthy that online data collection through structured questions with predefined answers affords significant operational advantages, such as national reach, efficiency in consolidating information, and lower logistical costs. However, this strategy has relevant limitations regarding the depth of collected information ^30^. The characterization of actions such as mandatory notifications, vaccination of priority groups, exams for people with chronic conditions, or active search for absentee users, although positively referred to by the teams, does not enable an accurate assessment of fundamental aspects such as periodicity, comprehensiveness of coverage, regularity of execution, or the degree of involvement of the different professionals in the reported practice. The assertion that a certain action is performed can mask significant variations in the frequency, consistency, and technical responsibility of its execution. As an alternative, and from the perspective of institutionalizing the evaluation, it should be complemented with other evaluation strategies, such as interviews with health care professionals and users that enable contextualizing the responses, capturing nuances of the work process, identifying operational barriers, and assessing the understanding of the actual functioning of PHCT. These recommendations do not diminish the capacity of the census results to provide elements that support health care policies in our country. Furthermore, the adopted design did not include the necessary and essential input from users, requiring the implementation of other evaluation strategies to address this significant deficiency.

### Ethical aspects

The census study was approved by the Research Ethics Committee of the School of Public Health, University of São Paulo (CAAE 78767024.2.0000.5421 and opinion 6.779.222). 

## Discussion

Although surprising, the maximum response rate resulted from an exhaustive and well-planned process of mobilizing municipal managers to join the census and professionals to fill out the questionnaire ^27^ ^,^ ^28^. This engagement shows the interest expressed by the BHU workers, who recognized in the census an opportunity to give visibility to the real working and structural conditions of the BHU, actively contributing to the production of strategic information for supporting PHC. It can be stated that the established governance structure, which coordinated federal, regional and local spheres and academia, facilitated the coordination of these initiatives, promoting integration between different government spheres and actors, and boosting institutional capacity for conducting the census.

Thus, the BHU Census contributed to the resumption of a systematic and permanent assessment movement in the SUS, overcoming the discontinuity caused by the termination of successful initiatives such as PMAQ-AB. The construction of a policy for regular and permanent PHC monitoring and assessment in Brazil has been reinforced by studies that emphasize the integration of the SUS basic network into territories that have been historically marked by deep social inequalities, which modulate the effectiveness of health care actions and programs ^14^ ^,^ ^16^. Studies with these characteristics are essential to overcome the normative nature of assessment, turning it into a valuable tool for management. By establishing a situational diagnosis of the PHC network, the BHU Census defined a baseline capable of providing robust evidence to support assessment research in explaining observed changes and inequalities arising from the social context and the installed capacity of the services. However, the comparison of the census results with findings from sample-based studies should consider the differences in the sampling plan (margin of error), the representativeness and scope of the studies (national versus local studies), and differences in the definition of concepts and operationalization of the variables studied.

The BHU Census represented a milestone for PHC assessment in Brazil, signifying the resumption of a national assessment policy after a period of discontinuity. It represents the SUS potential to be an important tool for coordinating and (re)activating knowledge production networks, integrating universities, managers, services, and social control in a proactive agenda for knowledge production on the situation of PHC in the SUS. It also provides methodological elements that contribute to the implementation of future assessment initiatives in Brazilian and international PHC.

It should be noted that resuming a policy agenda in the field of PHC monitoring and assessment - its institutionalization - requires not only data collection but also the construction of continuous participatory processes involving the academia, managers, health care professionals, and social control. 

The BHU Census, in addition to providing numerous inputs for health care policies, contributes to the consolidation of a national assessment and monitoring policy, guided by the PHC and SUS principles. 

In summary, the BHU Census represents a significant advance in the institutionalization of PHC assessment in Brazil. The data collection scope, the methodological rigor employed in its design and implementation, and the broad participation of the actors involved give the study an unparalleled potential to provide a detailed overview of the situation of PHC across the country. The census results provide evidence that can guide the formulation of more effective, equitable public policies aligned with the SUS principles, contributing toward improved PHC effectiveness and quality for the benefit of the entire Brazilian population.

## Data Availability

The research data are available upon request to the corresponding author.
