# Supplementary material for: Primary health care assessment in Brazil: conception and methodology of the 2024 National Basic Health Unit Census
Source: Cad Saude Publica. 2026 Apr 10;42:e00164625. doi: 10.1590/0102-311XEN164625 (PMC13078778; doi:10.1590/0102-311XEN164625)
Supplement: Translation

# Translation: Avaliação da atenção primária à saúde no Brasil: concepção e metodologia do Censo Nacional das Unidades Básicas de Saúde 2024

**DOI:** 10.1590/0102-311XPT164625

**Authors:** Aylene Bousquat, Ligia Giovanella, Rosana Aquino, Ana Luiza Queiroz Vilasbôas, Elaine Thumé, Elaine Tomasi, Maria Helena Magalhães de Mendonça, Alaneir de Fátima da Silva, Patty Fidelis de Almeida, Paulo Henrique dos Santos Mota, Carlos Pilz, Elisandréa Sguario Kemper, Dirceu Klitzke, Gabriela da Silva Formoso, Bruna Venturin, Luiz Augusto Facchini

**Affiliations:** 1 Faculdade de Saúde Pública, Universidade de São Paulo, São Paulo, Brasil.; 2 Centro de Estudos Estratégicos da Fiocruz Antonio Ivo de Carvalho, Fundação Oswaldo Cruz, Rio de Janeiro, Brasil.; 3 Instituto de Saúde Coletiva, Universidade Federal da Bahia, Salvador, Brasil.; 4 Faculdade de Enfermagem, Universidade Federal de Pelotas, Pelotas, Brasil.; 5 Faculdade de Medicina, Universidade Federal de Pelotas, Pelotas, Brasil.; 6 Escola Nacional de Saúde Pública Sergio Arouca, Fundação Oswaldo Cruz, Rio de Janeiro, Brasil.; 7 Faculdade de Medicina, Universidade Federal de Minas Gerais, Belo Horizonte, Brasil.; 8 Universidade Federal Fluminense, Niterói, Brasil.; 9 Secretaria de Atenção Primária à Saúde, Ministério da Saúde, Brasília, Brasil.; 10 Centro de Integração de Dados e Conhecimentos para Saúde, Salvador, Brasil.

**Keywords:** Atenção Primária à Saúde, Avaliação em Saúde, Serviços de Saúde, Pesquisa sobre Serviços de Saúde

## Abstract

A atenção primária à saúde (APS) é a base do Sistema Único de Saúde (SUS). Após um período de descontinuidade nas políticas de avaliação da APS, o Ministério da Saúde, com o apoio da Rede de Pesquisa em APS da Associação Brasileira de Saúde Coletiva (Abrasco), realizou o Censo Nacional das Unidades Básicas de Saúde em 2024. O objetivo do presente artigo é apresentar sua concepção e operacionalização. A metodologia do censo foi pautada em uma abordagem participativa, envolvendo diversos atores, incluindo secretarias do Ministério da Saúde, Conselho Nacional de Secretários de Saúde, Conselho Nacional de Secretarias Municipais de Saúde, instituições acadêmicas e Conselho Nacional de Saúde. O instrumento de coleta de dados foi construído de forma colaborativa e aplicado de forma online, utilizando a plataforma e-Gestor APS para garantir a institucionalização e segurança dos dados. A operacionalização contou com uma ampla rede de apoio em todos os estados, com o intuito de mobilizar gestores e profissionais. O censo obteve adesão de 100% dos municípios, com respostas de todas as 44.938 unidades básicas de saúde (UBS) do país. Esse sucesso, apesar dos desafios metodológicos inerentes a inquéritos online, demonstra o engajamento dos atores envolvidos no fortalecimento do SUS. O processo de elaboração do Censo das UBS 2024 engloba elementos que o qualificam para contribuir para a formulação de políticas públicas mais eficazes, equitativas e alinhadas aos princípios da APS e do SUS, contribuindo significativamente para a institucionalização da avaliação da APS no Brasil.

## Introdução

A atenção primária à saúde (APS) é reconhecida internacionalmente como a base dos sistemas de saúde, sendo fundamental para garantir o acesso universal, a coordenação do cuidado e a integralidade das ações ^1^ ^,^ ^2^. No Brasil, a Estratégia Saúde da Família (ESF) consolidou-se, ao longo de três décadas, como o principal modelo de organização da APS, com impacto positivo sobre a redução de desigualdades em saúde e melhoria de indicadores de morbimortalidade. Avaliações da APS brasileira realizadas nas últimas décadas demonstram que esta vem contribuindo para a consecução dos princípios constitucionais do Sistema Único de Saúde (SUS) - universalidade, integralidade e equidade ^3^ ^,^ ^4^ ^,^ ^5^ ^,^ ^6^ ^,^ ^7^.

No cenário internacional, a avaliação da APS vem orientando a construção de políticas públicas e promovendo a qualidade do cuidado. Destacam-se iniciativas como o *Quality and Outcomes Framework* (QOF, Quadro de Qualidades e Resultados), implementado no Reino Unido desde 2004, que estabeleceu mecanismos de pagamento por desempenho e indicadores padronizados de qualidade ^8^. Na região das Américas, a Organização Pan-Americana da Saúde (OPAS), em parceria com o Banco Mundial, lidera o *Primary Health Care Measurement and Improvement Initiative* (PHCMI, Iniciativa de Medição e Melhoria da Atenção Primária à Saúde), que visa padronizar e fortalecer os sistemas de avaliação da APS nos países membros ^9^.

No Brasil, país com dimensão continental e com a maior rede pública de serviços de APS no mundo, a avaliação da APS não é tarefa trivial, exigindo modelos participativos e inovadores. Ademais, a concepção ampliada da APS brasileira - não restrita ao primeiro nível de cuidado ambulatorial, mas estruturada em forte dimensão comunitária e territorial, e com funções como a de coordenadora do cuidado, ao acompanhar continuamente a trajetória clínica de cada pessoa, e como ordenadora da rede de atenção, ao organizar fluxos e regular o acesso entre os diferentes serviços do SUS - adiciona novos e necessários desafios avaliativos ^1^ ^,^ ^10^ ^,^ ^11^.

Entendendo a necessidade de fortalecer a avaliação da APS brasileira, o Ministério da Saúde, por meio da Secretaria de Atenção Primária à Saúde (SAPS), com o apoio da Rede de Pesquisa em APS da Associação Brasileira de Saúde Coletiva (Rede APS/Abrasco), realizou o Censo Nacional das Unidades Básicas de Saúde (Censo das UBS) em 2024. O Censo das UBS marca a retomada de uma política de avaliação da APS para todas as unidades básicas de saúde (UBS), após um período de 12 anos. Cabe destacar que, no levantamento censitário realizado em 2012, no seio do Programa de Melhoria do Acesso e da Qualidade da Atenção Básica (PMAQ-AB), foram colhidas apenas informações de estrutura ao conjunto das UBS. Portanto, o formato atual do Censo das UBS de 2024 - que integra dados sobre infraestrutura física e a oferta de ações e serviços da APS - representa um processo inédito na abrangência e caracterização das UBS brasileiras. Além de atualizar a base de dados sobre a rede de serviços de APS e suas condições de funcionamento, o censo teve como objetivo fornecer informações capazes de orientar as políticas públicas de saúde, subsidiando não apenas decisões pontuais, mas também contribuindo para uma política nacional de avaliação e monitoramento. Seu delineamento foi pautado pelos princípios do SUS, em favor da universalização e qualificação da ESF, permitindo a análise detalhada da situação da APS do país. O presente artigo tem como objetivo apresentar a concepção e operacionalização do Censo das UBS realizado em 2024. O processo de elaboração do censo partiu de dois enfoques complementares e indissociáveis: (1) a garantia de um processo que dialogasse com a estrutura federativa brasileira e (2) a definição da imagem-objetivo da APS brasileira, entendida como integral, resolutiva, comunitária, territorial e plenamente integrada à rede de serviços de saúde. 

## O Censo das UBS de 2024 e a avaliação da APS brasileira

Nas últimas três décadas, o governo brasileiro implementou diversas iniciativas para monitorar e avaliar o desempenho da APS, com especial ênfase na ESF. Em 1998, foi instituído o Sistema de Informação da Atenção Básica (SIAB), para o acompanhamento rotineiro das atividades das equipes. Neste mesmo ano, foi criado o Pacto de Indicadores da Atenção Básica, consolidando nacionalmente instrumentos específicos para monitoramento da APS. Em 2002, o lançamento do Projeto de Expansão e Consolidação da Saúde da Família (PROESF) trouxe avanços significativos para a avaliação e monitoramento, incluindo o estímulo a pesquisas avaliativas e, em 2005, os Estudos de Linha de Base estabeleceram os indicadores de desempenho iniciais ^12^. 

Em 2006, a Política Nacional de Atenção Básica (PNAB) incorporou, de forma inédita, um processo formal de pactuação interfederativa de metas, por meio do Pacto de Indicadores da Atenção Básica, como estratégia para monitoramento do desempenho da APS e indução à melhoria da qualidade do cuidado. Esse pacto previa a definição e o acompanhamento anual de metas em indicadores de saúde previamente acordados entre os entes federativos, em conformidade com o Pacto pela Saúde. O cumprimento dessas metas era vinculado à correção anual dos valores do Piso da Atenção Básica Fixo (PAB Fixo), estabelecendo, assim, uma relação direta entre desempenho e financiamento federal. Essa iniciativa representou um marco na institucionalização da avaliação do desempenho da atenção básica no Brasil, promovendo maior corresponsabilidade entre os níveis de gestão e incentivando a consolidação da APS como política pública estratégica no SUS. Neste mesmo período, os Pactos pela Saúde introduziram novos parâmetros para a gestão e avaliação das equipes, seguidos, em 2011, pela implementação da avaliação para melhoria da qualidade, que passou a mensurar aspectos qualitativos e organizacionais das equipes de ESF. 

O PMAQ-AB foi outro processo importante na avaliação na APS no país ^13^. Em 2012, no âmbito do PMAQ-AB, ocorreu o primeiro Censo Nacional de Estrutura das UBS, fornecendo um diagnóstico abrangente das condições estruturais das unidades ^14^. Ciclos subsequentes de avaliação do PMAQ-AB foram realizados nos períodos de 2012-2014, 2015-2016 e 2017-2018, tendo como unidade de análise as equipes de saúde, sobretudo as equipe de saúde da família (eSF), equipe de saúde bucal (eSB) e Núcleo de Apoio à Saúde da Família (NASF).

A coleta periódica de dados sobre infraestrutura, práticas assistenciais, organização do trabalho e satisfação dos usuários ocorrida nesse período gerou um acervo abrangente de informações que subsidiou decisões estratégicas nas políticas públicas nacionais ^15^ ^,^ ^16^.

O período posterior à realização do último ciclo do PMAQ-AB (2017-2018) foi marcado por: uma crescente fragilização institucional nas políticas de saúde; descontinuidades de programas; desestruturação de equipes técnicas, e; perda de mecanismos federativos de indução e monitoramento em geral e particularmente da APS ^10^ ^,^ ^11^. Em 2019, os processos avaliativos anteriores foram substituídos pela proposta de monitoramento quadrimestral de sete indicadores de desempenho das equipes de saúde, dentro do componente de pagamento por desempenho do programa Previne Brasil. O Previne Brasil foi uma solução de continuidade precária e restrita na política até então vigente de avaliação da APS, contribuindo para o desmonte da avaliação da APS e de sua institucionalização ^17^ ^,^ ^18^.

O Censo das UBS, realizado em 2024, marca a retomada de uma política nacional de avaliação e monitoramento, pautada pelos princípios da APS e do SUS, contribuindo para a institucionalização da avaliação no SUS, como será demonstrado a seguir.

Na Figura 1, é possível visualizar a linha do tempo dos principais marcos do monitoramento e avaliação de políticas de saúde entre 1996 e 2025.

**Figura 1:** Principais marcos de monitoramento e avaliação de políticas públicas de saúde no Brasil.

PMAQ-AB: Programa de Melhoria do Acesso e da Qualidade da Atenção Básica. Fonte: elaboração própria.

## Metodologia

Para descrever o caminho metodológico realizado serão descritos os seguintes passos: governança do censo; definição do universo; elaboração e validação do questionário; coleta dos dados; estratégias para operacionalização do censo; participantes do censo e análise de completude do banco; limitações, e; aspectos éticos.

### Governança do censo

O desenvolvimento metodológico do Censo das UBS foi fundamentado na abordagem participativa da avaliação de quarta geração proposta por Guba & Lincoln ^19^. Esse modelo enfatiza a participação ativa de diferentes grupos interessados na construção do instrumento avaliativo, privilegiando a negociação de significados e a construção coletiva das dimensões e indicadores do estudo. Tal abordagem foi adotada visando garantir a relevância, legitimidade e representatividade dos aspectos avaliados, considerando as especificidades regionais e as necessidades concretas das equipes e gestores locais da APS brasileira. 

Nesta direção, para além da SAPS e da Rede APS/Abrasco, o processo incluiu ativamente: outras secretarias do Ministério da Saúde; o Conselho Nacional de Secretários de Saúde (CONASS), representando as secretarias estaduais de saúde; o Conselho Nacional de Secretarias Municipais de Saúde (CONASEMS),representando as secretarias municipais de saúde; instituições acadêmicas e de pesquisa; entidades representativas de profissionais; organizações internacionais, como a OPAS, e; o Conselho Nacional de Saúde (CNS), representando o controle social e a participação comunitária. 

### Definição do universo

Apesar da existência de Cadastro Nacional de Estabelecimentos de Saúde (CNES), a definição do universo do estudo não foi trivial. A meta era que todas as unidades de saúde que oferecessem cuidado de APS fossem incluídas, o que excluía diversas unidades que oferecem cuidados ambulatoriais para grupos específicos, como por exemplo: atendimento de pediatria, ginecologia e que muitas vezes constavam como de APS no CNES. Ademais, diversas unidades que já foram UBS mudaram suas atividades, mas continuavam cadastradas como de APS. Dessa forma, para chegar ao universo foram realizados os seguintes passos sequenciais: (i) inclusão de todas as unidades que constavam como de APS no CNES em abril de 2024; (ii) validação pelos gestores municipais; (iii) checagem das respostas do censo.

### Elaboração e validação questionário

O instrumento preliminar, elaborado pela equipe de pesquisadores da Rede APS/Abrasco, foi orientado pela concepção de APS com orientação comunitária, territorializada, de qualidade, efetiva, integrada à rede regionalizada de atenção à saúde do SUS ^20^ entre maio e setembro de 2023. Esta abordagem abrangente sustenta-se em equipes multiprofissionais com trabalho interprofissional e colaborativo; enfoque territorial e comunitário; vigilância em saúde e responsabilização pela saúde da população de determinado território; participação social e intersetorialidade; escopo ampliado de ações individuais e coletivas; coordenação do cuidado e integração à rede de saúde; abordagem intercultural; promoção da equidade, e; valorização da força de trabalho. Todos estes aspectos foram incluídos no questionário. A equipe que elaborou a primeira versão do instrumento era composta por onze pesquisadores sêniores com larga experiência na avaliação da APS. Sempre que pertinente, foram resgatadas questões do PMAQ-AB o que permitiria, com os devidos cuidados metodológicos, comparações. Para garantir que o instrumento final fosse capaz tanto de refletir as necessidades quanto as especificidades da APS no país, bem como planejar a sua operacionalização, foram realizadas diversas oficinas e reuniões técnicas, que contaram com a participação dos inúmeros envolvidos, desde a primeira versão do questionário. Entre setembro de 2023 e fevereiro de 2024, ocorreram quatro oficinas nacionais presenciais que se debruçaram sobre o instrumento inicial, realizando adequações até a conclusão do instrumento que seria objeto de pré-teste. Reuniões técnicas semanais para aprimoramento do instrumento foram realizadas entre setembro de 2023 e março de 2024 e serviram como espaços de pactuação de consensos.

Durante a última oficina nacional presencial, os participantes identificaram os principais desafios para a implementação do censo e propuseram soluções para superá-los. Entre os desafios, destacavam-se a necessidade de garantir a confiabilidade e objetividade do instrumento, mobilizar e engajar os atores envolvidos, superar gargalos tecnológicos, considerar aspectos políticos e temporais, assegurar o sigilo e a ética na pesquisa. 

As propostas para superar esses desafios envolveram a revisão e aperfeiçoamento do instrumento de coleta de dados e a elaboração do manual de orientação de preenchimento do instrumento, desenvolvimento de estratégias de comunicação e mobilização, capacitação dos respondentes, implementação de apoio técnico e logístico, além do planejamento de um cronograma adequado.

Finalmente, o instrumento do Censo das UBS, proposto após as rodadas das oficinas e reuniões técnicas, foi submetido a um processo de validação que incluiu nova revisão por especialistas, testes piloto e pactuação tripartite. Os testes pilotos ocorreram inicialmente com o questionário em papel em seis UBS escolhidas para se analisar especialmente o entendimento das perguntas por parte dos respondentes, bem como observar sua exequibilidade em distintos tipos de UBS, como por exemplo, com e sem ESF. Em maio de 2024, novo teste piloto foi realizado já com o instrumento online em UBS em três estados da federação, para que fossem identificadas dificuldades no uso da plataforma. Após os dois testes, foram realizados pequenos ajustes que não modificaram substancialmente o instrumento.

O questionário final aglutinou 15 dimensões e 141 questões, sendo 140 em formato estruturado com opções de resposta em formato sim/não, escala Likert e em menor número ordinais, além uma questão aberta incluída ao final do instrumento como opcional para o respondente tecer os comentários que desejasse. Cada dimensão foi dividida em componentes específicos, abrangendo informações detalhadas sobre infraestrutura física, organização e oferta de serviços, práticas assistenciais, coordenação do cuidado, incorporação tecnológica, gestão e processo de trabalho das equipes, ações comunitárias e intersetoriais, vigilância em saúde, e medidas voltadas à promoção da equidade, à valorização profissional, entre outros (Quadro 1). Para garantir o diálogo com as distintas características regionais foram incluídas questões referentes a equipes ribeirinhas, bem como populações e povos tradicionais. Mais informações e detalhes das dimensões e variáveis estão disponíveis no questionário ^21^ e no manual instrutivo ^22^ do Ministério da Saúde.

**Quadro 1:** Dimensões e componentes avaliadas no Censo Nacional das Unidades Básicas de Saúde, 2024.

DIMENSÃO	COMPONENTES
1. Identificação da UBS	Dados de localização, funcionamento e gestão administrativa
2. Composição das equipes de APS, gestão e processo de trabalho	Tipos e número de equipes; profissionais que atuam na UBS; processo de trabalho das equipes; tipos de vínculos e contratos de trabalho
3. Condições de infraestrutura da UBS	Estrutura física e ambiência; acessibilidade; disponibilidade de equipamentos e insumos; construção, ampliação e reformas
4. Saúde digital: incorporação de tecnologias digitais e telessaúde	Equipamentos de tecnologia da informação e acesso à internet; telessaúde; uso de prontuário eletrônico
5. Oferta e acesso a métodos diagnósticos e medicamentos	Realização de exames e testes diagnósticos; acesso a medicamentos
6. Oferta de ações e serviços na UBS: escopo das práticas das equipes	Saúde sexual e reprodutiva; atenção ao pré-natal e puerpério; detecção, prevenção e rastreio de câncer do colo do útero e mama; atenção à criança até dois anos; atenção à pessoa com hipertensão arteriar sistêmica; atenção à pessoa com diabetes mellitus; atenção à pessoa com sobrepeso/obesidade; atenção à pessoa com tuberculose; atenção à pessoa com hanseníase; atenção em saúde mental; atenção à pessoa em situação de violência; atenção à saúde das pessoas idosas; atenção integral à saúde do homem; atenção às pessoas acamadas; incentivo à universalização da cobertura vacinal; atenção às condições pós-COVID-19; práticas integrativas e complementares (PICS); atenção às urgências e emergências
7. Promoção da saúde e ações intersetoriais	Ações e programas de promoção da saúde; Programa Academia da Saúde; Programa Saúde na Escola (PSE); Programa Bolsa Família e ações intersetoriais
8. Vigilância em saúde	Atividades de vigilância epidemiológica; vigilância alimentar e nutricional; vigilância sanitária e ambiental; combate ao *Aedes aegypti*
9. Equipes de saúde bucal: atuação e escopo de práticas	Organização da agenda e oferta das ações da equipe; relação da equipe de saúde bucal com a rede de atenção; atenção ao câncer de boca
10. Atuação dos agentes comunitários de saúde	Atividades específicas realizadas pelos ACS
11. Coordenação do cuidado e integração à rede de atenção	Organização da agenda, acolhimento e demanda espontânea; integração da APS com outros pontos da rede; regulação assistencial e transporte sanitário
12. Cuidado compartilhado e atuação das equipes multiprofissionais na APS	Composição e escopo das equipes multiprofissionais
13. Ações comunitárias e territoriais	Territorialização e população de referência; participação social e estratégias de comunicação com usuários
14. Promoção da equidade	Atenção às populações tradicionais e grupos específicos; atenção à pessoa com deficiência; ações de promoção de equidade de gênero, orientação sexual, étnica e racial
15. Educação permanente, qualificação e valorização dos trabalhadores	Ações relacionadas à saúde dos trabalhadores e gestão do trabalho

ACS: agentes comunitários de saúde; APS: atenção primária à saúde; UBS: unidades básicas de saúde.

Fonte: elaboração própria.

### Coleta dos dados

Tomando por base as experiências de pesquisas prévias conduzidas pela Rede APS/Abrasco durante a pandemia da COVID-19 ^23^ ^,^ ^24^, optou-se pelo formato online para a coleta de dados. Ressalta-se que inquéritos online agregam as vantagens de serem mais ágeis e de demandarem volumes financeiros menores do que versões presenciais, qualidades estas extremamente desejáveis quando se trata da administração pública. 

Para facilitar e garantir a institucionalização do processo, optou-se pelo uso dos sistemas de informação oficiais do Ministério da Saúde, que, além de garantir a segurança no tráfego dos dados, é de uso rotineiro das secretarias municipais e das UBS. Dessa forma, foi utilizado o e-Gestor Atenção Primária à Saúde (e-Gestor APS), uma plataforma informatizada desenvolvida pela SAPS destinada à gestão de cadastros de estabelecimentos, equipes e profissionais, bem como ao acompanhamento de programas e financiamentos federais no âmbito da APS. O acesso ao sistema foi realizado por meio de autenticação via conta gov.br, mecanismo oficial de identificação digital do Governo Federal, conforme previsto nos protocolos de acesso do próprio sistema e-Gestor APS ^25^. Essa configuração assegurava identificação nominal dos usuários e rastreabilidade das ações realizadas na plataforma, garantindo a integridade dos registros. No âmbito do Censo das UBS, o e-Gestor APS foi utilizado como ambiente operacional exclusivo para a gestão do censo. 

Inicialmente, o gestor municipal deveria indicar sua decisão de participar do Censo das UBS, mediante funcionalidade específica disponibilizada na plataforma Gerencia APS, sendo necessárias habilitação e autenticação prévias. Na sequência, ocorria o cadastramento e designação do responsável municipal pelo Censo das UBS. Após a adesão, o Gestor Municipal acessava uma lista na qual constavam os estabelecimentos de saúde de APS a partir dos dados disponíveis no CNES em abril de 2024. Caso algum destes estabelecimentos não estivesse mais funcionando, ou não fosse mais realmente um serviço de APS, este deveria ser inativado. Para cada um dos serviços ativados, o responsável do censo indicaria um respondente para preencher o questionário do censo.

Após a indicação do responsável pelo preenchimento do questionário do censo, o instrumento de coleta era disponibilizado eletronicamente por meio da plataforma LimeSurvey (https://www.limesurvey.org/pt-br), hospedada na infraestrutura do Ministério da Saúde e integrada ao e-Gestor APS. 

O processo de adesão e posterior preenchimento dos questionários teve início em 03 de junho de 2024 e foi concluído em 30 de setembro de 2024, com prorrogação específica para municípios do Rio Grande do Sul devido a eventos climáticos excepcionais enfrentados pela população do estado. Dessa forma, os resultados do censo se referem ao perfil das UBS entre junho e setembro de 2024.

### Estratégias para operacionalização do censo

Para garantir a adesão e respostas, foi estruturada uma ampla rede de apoio composta por equipes específicas em cada um dos estados, que era responsável pela articulação institucional, planejamento estratégico das ações locais e supervisão das atividades, assim como apoio para a coleta de dados. A equipe de cada estado, organizada pela Rede APS/Abrasco, contava com: um(a) pesquisador(a) sênior; um a quatro articuladores/facilitadores, além de um a 12 animadores. A constituição das equipes de trabalho de campo em cada estado visou favorecer a comunicação e desenvolver estratégias para a adesão e participação dos gestores municipais, gerentes locais e profissionais das UBS no preenchimento do instrumento do censo, desenvolvendo atividades de articulação, cooperação e mobilização refletindo a concepção de governança adotada.

Os articuladores estaduais do censo estabeleceram contatos institucionais e promoveram reuniões periódicas, bem como trabalho conjunto com representantes das Secretarias Estaduais de Saúde (SES), dos Conselhos de Secretarias Municipais de Saúde (COSEMS) e das Superintendências Estaduais do Ministério da Saúde, assegurando o suporte institucional necessário para a execução do censo. Ademais, participaram regularmente das reuniões de Comissões Intergestores Bipartite (CIB) e de Comissões Intergestores Regionais (CIR), nas quais formalizaram pactuações essenciais para a ampla adesão ao censo.

Os animadores tiveram a atribuição de realizar contatos diretos e sistemáticos com gestores municipais e equipes de saúde das UBS, assegurando a adesão ao censo e esclarecendo dúvidas operacionais relacionadas ao preenchimento do questionário. Os contatos foram predominantemente realizados via telefone, aplicativos de mensagens, e-mails institucionais e videoconferências. Em casos de dificuldades para a adesão ou no processo de resposta ao censo, foram promovidas pelos animadores reuniões online e/ou presenciais para prestar suporte direto e resolver entraves operacionais. Pelo grande volume de dados, em vários momentos, ocorreu instabilidade da plataforma, o que demandava um suporte maior às equipes das UBS. Para gerenciamento e monitoramento das atividades diárias, cada equipe estadual construiu instrumentos próprios, destacando-se a utilização de planilhas compartilhadas. Esses documentos registravam diariamente as tentativas de contato, o status detalhado da adesão municipal, a identificação dos profissionais locais responsáveis pelas respostas e quaisquer dificuldades encontradas. As equipes recebiam diariamente relatórios gerados pela equipe do Ministério da Saúde com a situação de resposta de cada estabelecimento participante do censo em seu território. Tal organização permitiu um acompanhamento contínuo e intervenções rápidas e direcionadas pelos coordenadores estaduais sempre que necessário.

Os facilitadores estaduais, em ação conjunta com técnicos da SAPS, promoveram diversas iniciativas para integrar os atores. Em diversos estados ocorreram parcerias com COSEMS com articulada ação conjunta, incluindo seus apoiadores, para divulgação do censo e incentivo à adesão. Em parceria com as coordenações estaduais da APS das SES, foram acionados apoiadores estaduais da APS; apoiadores regionais das SES nas CIR com participação de facilitadores estaduais e animadores nas reuniões das CIR. Este conjunto de estratégias e atores ajudaram a viabilizar o censo.

Como estratégia complementar de mobilização e esclarecimento, foram promovidos eventos digitais informativos, tais como lives e webinários, direcionados especialmente para gestores municipais e equipes das UBS. Esses eventos tinham o objetivo específico de sensibilizar os participantes sobre a relevância do estudo e oferecer orientações detalhadas sobre os procedimentos metodológicos e operacionais de preenchimento do questionário.

Na perspectiva de ampliar as possibilidades de esclarecimento de dúvidas de forma mais interativa, foram disponibilizados materiais de apoio como manuais e vídeos instrutivos que detalharam o passo a passo para manifestação de interesse, cadastro dos respondentes e resposta ao censo. Os vídeos instrutivos e as lives nacionais realizadas pela Rede APS/Abrasco, Ministério da Saúde e CONASEMS abordavam diferentes momentos do censo e atingiram 29.500 visualizações, sem contar que houve algumas lives estaduais, como por exemplo a de São Paulo que chegou a 2.500 acessos. Reuniões técnicas online foram realizadas em todos os estados com a participação da SAPS, CONASS, CONASEMS, COSEMS, Rede APS/Abrasco, facilitadores e animadores.

Finalmente, para garantir rapidez nas decisões operacionais e comunicação efetiva entre as diversas equipes estaduais e a coordenação nacional do censo, foram organizados grupos institucionais por meio de aplicativo de mensagem. Esses grupos incluíam os coordenadores estaduais, representantes das SES, dos COSEMS e da equipe nacional do Ministério da Saúde. Essa ferramenta permitiu solução ágil de problemas operacionais, disseminação rápida de boas práticas identificadas em diferentes estados e reforçou o alinhamento metodológico em todo o território nacional.

Ainda na perspectiva de garantir suporte e esclarecimento sobre eventuais dúvidas ou dificuldades relacionadas à plataforma do censo, foi estruturado um ambiente de atendimento que contemplou os seguintes canais: o Disque Censo, por meio de WhatsApp e teleatendimento, disponível em horário comercial, um e-mail específico e um serviço de web atendimento do sistema e-Gestor APS. Após as duas primeiras semanas do início da coleta de dados do censo, foi elaborado um *Frequently Asked Questions* (FAQ, Dúvidas Frequentes) contendo respostas às principais dúvidas recebidas pelos canais de atendimento ^26^. Esses recursos ofereciam orientações ágeis e precisas aos gestores e profissionais de saúde, assegurando maior eficiência no preenchimento, garantindo que as dúvidas ou problemas verificados fossem respondidas de forma mais rápida possível.

### Participantes do censo e análise de completude do banco

O Censo das UBS teve caráter censitário, transversal e descritivo e teve adesão de 100% dos gestores municipais. Inicialmente, foram incluídos 49.738 estabelecimentos de saúde cadastrados no CNES na tipologia serviços de APS do SUS no mês de abril de 2024. Estes serviços aglutinavam mais de 90 mil equipes de saúde de APS, incluindo eSF, eAP (equipes de atenção primária), eSB, equipes multiprofissionais, além de outras modalidades de equipes, como, por exemplo, equipes de Consultório na Rua (eCR), equipe de atenção primária prisional (eAPP) e equipes de saúde da família ribeirinha (eSFR). Desse total, 4.026 estabelecimentos foram inativados pelos gestores municipais com a justificativa de que não estavam em funcionamento no momento de resposta ao censo, ou por se tratar de outro tipo de estabelecimento não referente à APS. A Figura 2 apresenta a evolução das respostas durante o período em que o questionário esteve disponível para preenchimento. 

**Figura 2:** Evolução das respostas ao censo durante o período de sua realização.

UBS: unidades básicas de saúde. Fonte: elaboração própria.

No total, 45.712 estabelecimentos de saúde responderam ao censo. O banco de dados foi analisado em sua completude e consistência, e observou-se que, mesmo após o processo de inativação, alguns estabelecimentos que não eram de APS haviam respondido ao questionário. Assim, foram excluídos da análise estabelecimentos que: (a) informaram, em questão aberta, que não eram de APS; (b) unidades móveis terrestres ou fluviais; (c) não possuíam financiamento do Ministério da Saúde para eSF, eAP, eSFR e agentes comunitários de saúde (ACS) nos 12 meses anteriores à realização do censo, e declararam não possuir eSF, eAP e eSFR, além de possuírem nomes claramente indicativos de estabelecimentos de outro nível de atenção (Centro Covid, Casas de Parto, Centros de Especialidades, Centro de Especialidades Odontológicas, Centro de Reabilitação), com a exclusão de 773 estabelecimentos (Figura 3).

**Figura 3:** Fluxograma do número de respostas obtidas pelo censo.

UBS: unidades básicas de saúde. * Unidades não possuíam financiamento do Ministério da Saúde para equipes de saúde da família (eSF), equipes atenção primária (eAP), equipes de saúde da família ribeirinha (eSFR) e agentes comunitários de saúde (ACS), e declararam não possuir eSF, eAP e eSFR, além de possuírem nomes claramente indicativos de estabelecimento de outro nível de atenção. Fonte: elaboração própria.

Desta forma, o universo foi de 44.938 UBS brasileiras, com a seguinte distribuição pelas regiões do país: Norte = 4.096 (9,11%), Nordeste = 17.737 (39,47%), Sudeste = 13.375 (29,76%), Sul = 6.607 (14,7%) e Centro-oeste = 3.213 (6,95%) estabelecimentos.

### Limitações

A literatura aponta para altas taxas de desistência ou de perda de respostas em estudos online, provocando viés de seleção e falta de representatividade da amostra ^27^ ^,^ ^28^. No caso do censo, evidentemente não havia um desenho amostral, mas perdas poderiam ter ocorridos pelos mais diversos motivos, desde dificuldades de conexão de internet até ausência de profissionais qualificados, levando a vieses de seleção. Este cenário não ocorreu e houve a superação destes desafios e a totalidade das UBS do país preencheram o instrumento do estudo. 

Apesar deste sucesso, não se pode deixar de mencionar as limitações e desafios metodológicos enfrentados. Após as primeiras etapas de análise de completude do banco de dados, foram identificadas algumas poucas questões com inconsistências nas respostas. Optou-se pela exclusão de tais variáveis, sendo elas: o horário de abertura e fechamento das UBS aos sábados e domingos e a questão referente à unidade respondente ser um Ponto de Apoio. Segundo o Ministério da Saúde ^29^, o Ponto de Apoio, apesar de ter um CNES próprio, está ligado funcionalmente a uma UBS com quem compartilha a equipe de saúde de forma periódica, de tal modo que suas atividades, especialmente as de profissionais de nível superior, não são diárias, mas isto não ficou claro para os respondentes. 

Vale ressaltar que a coleta de dados online por meio de perguntas estruturadas com respostas predefinidas oferece facilidades operacionais importantes, como abrangência nacional, agilidade na consolidação das informações e menor custo logístico. No entanto, essa estratégia apresenta limitações relevantes quanto à profundidade da informação coletada ^30^. A caracterização de ações como notificações compulsórias, vacinação de grupos prioritários, exames em pessoas com condições crônicas ou busca ativa de usuários faltosos, embora referida positivamente pelas equipes, não permite aferir com exatidão aspectos fundamentais como a periodicidade, a integralidade da cobertura, a regularidade da execução ou o grau de envolvimento dos diferentes profissionais na prática relatada. A afirmativa de que determinada ação é realizada pode encobrir variações significativas na frequência, na consistência e na responsabilidade técnica da execução. Como alternativa, e na perspectiva de institucionalização da avaliação, recomenda-se a complementação com outras estratégias de avaliação, como entrevistas com profissionais de saúde e usuários, que permitam contextualizar as respostas, captar nuances do processo de trabalho, identificar barreiras operacionais e qualificar a compreensão sobre o funcionamento real das equipes da APS. Estas recomendações não diminuem a capacidade dos resultados do censo em fornecer elementos que subsidiem as políticas de saúde em nosso país. Ademais, o desenho adotado não incluiu o olhar necessário e imprescindível dos usuários, cobrando a realização de outras estratégias avaliativas que sanem esta importante ausência.

### Aspectos éticos

O estudo do censo foi aprovado pelo Comitê de Ética em Pesquisa da Faculdade de Saúde Pública, Universidade de São Paulo (CAAE 78767024.2.0000.5421 e parecer 6.779.222). 

## Discussão

Embora surpreendente, a taxa máxima de resposta decorreu de um exaustivo e bem planejado processo de mobilização de gestores municipais para adesão ao censo e dos profissionais para preenchimento do questionário ^27^ ^,^ ^28^. Esse engajamento expressa o interesse demonstrado pelos trabalhadores e trabalhadoras das UBS, que reconheceram no censo uma oportunidade de dar visibilidade às condições reais de trabalho e de estrutura das UBS, contribuindo ativamente com a produção de informações estratégicas para o fortalecimento da APS. Pode-se afirmar que a estrutura de governança estabelecida, que articulava os três níveis federados e a academia, facilitou a coordenação dessas iniciativas, promovendo a integração entre os diferentes atores e níveis de gestão, e fortalecendo a capacidade institucional para a condução do censo.

Assim, o Censo das UBS contribuiu para a retomada de um movimento avaliativo sistemático e permanente no SUS, superando a descontinuidade ocasionada pelo fim de iniciativas exitosas como o PMAQ-AB. A construção de uma política de monitoramento e avaliação regular e permanente para a APS no Brasil tem sido reforçada por investigações que enfatizam a inserção da rede básica do SUS em territórios historicamente marcados por profundas desigualdades sociais, que modulam a efetividade das ações e programas de saúde ^14^ ^,^ ^16^. Estudos com estas características são indispensáveis para superar o caráter normativo da avaliação, convertendo-a em uma ferramenta valiosa para a gestão. Ao estabelecer um diagnóstico situacional da rede básica, o Censo das UBS definiu uma linha de base capaz de fornecer evidências robustas para apoiar pesquisas avaliativas na explicação das mudanças observadas e das desigualdades decorrentes do contexto social e da capacidade instalada dos serviços. No entanto, a comparação dos resultados do censo com achados de pesquisas de base amostral deve considerar as diferenças no plano amostral (margem de erro das estimativas), a representatividade e abrangência dos estudos (estudos nacionais *versus* locais) e diferenças na definição de conceitos e operacionalização das variáveis estudadas.

O Censo das UBS representou um marco para a avaliação da APS no Brasil, significando a retomada de uma política nacional de avaliação após um período de descontinuidade. Representa o potencial do SUS em ser um importante dispositivo para articular e (re)ativar redes de produção de conhecimento, integrando universidades, gestores, serviços e o controle social em uma agenda propositiva e de produção de conhecimento sobre a situação da APS no SUS. Fornece também elementos metodológicos que contribuem para a realização de futuras iniciativas de avaliação na APS brasileira e internacional.

Cabe ressaltar que a retomada de uma agenda de políticas no campo do monitoramento e da avaliação na APS - sua institucionalização - exige não apenas a coleta de dados, mas também a construção de processos participativos contínuos que envolvam a academia, gestores, profissionais de saúde e o controle social. 

O Censo das UBS, além de fornecer inúmeros subsídios para as políticas de saúde, contribui para a consolidação de uma política nacional de avaliação e monitoramento, pautada pelos princípios da APS e do SUS. 

Em suma, o Censo das UBS representa um avanço significativo no processo de institucionalização da avaliação da APS no Brasil. A abrangência da coleta de dados, o rigor metodológico empregado em sua concepção e operacionalização, e a ampla participação dos atores envolvidos conferem ao estudo um potencial ímpar para fornecer um panorama detalhado da situação da APS em todo o país. Os resultados do censo trazem evidências que podem orientar a formulação de políticas públicas mais efetivas, equitativas e alinhadas aos princípios do SUS, contribuindo para o fortalecimento e a qualificação da APS em benefício de toda a população brasileira.

## Data Availability

Os dados de pesquisa estão disponíveis mediante solicitação ao autor de correspondência.
